# Does Citizen Engagement With Government Social Media Accounts Differ During the Different Stages of Public Health Crises? An Empirical Examination of the COVID-19 Pandemic

**DOI:** 10.3389/fpubh.2022.807459

**Published:** 2022-06-13

**Authors:** Wei Zhang, Hui Yuan, Chengyan Zhu, Qiang Chen, Richard Evans

**Affiliations:** ^1^School of Medicine and Health Management, Huazhong University of Science and Technology, Wuhan, China; ^2^School of Political Science and Public Administration, Wuhan University, Wuhan, China; ^3^School of Journalism and New Media, Xi'an JiaoTong University, Xi'an, China; ^4^Faculty of Computer Science, Dalhousie University, Halifax, NS, Canada

**Keywords:** government social media, citizen engagement, public health crisis, crisis stage, dialogic communication, social media capital, information richness, language features

## Abstract

**Background:**

The COVID-19 pandemic has created one of the greatest challenges to humankind, developing long-lasting socio-economic impacts on our health and wellbeing, employment, and global economy. Citizen engagement with government social media accounts has proven crucial for the effective communication and management of public health crisis. Although much research has explored the societal impact of the pandemic, extant literature has failed to create a systematic and dynamic model that examines the formation mechanism of citizen engagement with government social media accounts at the different stages of the COVID-19 pandemic. This study fills this gap by employing the Heuristic-Systematic Model and investigating the effects of the heuristic clues including social media capital, information richness, language features, dialogic loop, and the systematic clue including content types, on citizen engagement with government social media across three different stages of the pandemic, employing the moderating role of emotional valence.

**Methods:**

The proposed model is validated by scraping 16,710 posts from 22 provincial and municipal government micro-blog accounts in the Hubei province, China.

**Results:**

Results show that the positive effects of social media capital on citizen engagement were observed at all stages. However, the effects of information richness, language features, dialogic loop, and content types, and the moderating effect of emotional valence, varied across the different pandemic development stages.

**Conclusions:**

The findings provide suggestions for the further effective use of government social media, and better cope with crises. Government agencies should pay attention to the content and form of information shared, using technical means to analyze the information needs of citizens at different stages of public health emergencies, understanding the content most concerned by citizens, and formulating the content type of posts.

## Introduction

Strong communication by governments during public health crises is essential for instilling trust in citizens and promoting two-way engagement, ultimately reducing societal fear, and uncertainty. Social media provides a mechanism for governments to clearly share their goals and response activities, keeping citizens well-informed and guiding response behaviors (1). Effective crisis communication delivers important crisis-related information (e.g., latest news, government reactions, advised responsive behaviors etc.) to targeted groups in a timely and effective fashion, increasing citizens' perception toward governmental transparency and capabilities (2). However, crisis communication fails if reliant on one-way information delivery. Instead, citizens are encouraged to participate during the entire crisis response process to maintain dialogue with governments.

During global health crises, such as COVID-19, government communication must encourage active public participation and attach significant attention to citizens' demands and expectations, while keeping information transparent and inclusive (3). If citizens do not participate and comply with government's responses, communication during the crisis can be deemed a failure, and government responses and emergency management are likely to face further challenges (4). Globally, it is observed that citizen engagement is the key to effective crisis communication (5, 6). In the social media era, this is of vital importance. Social media has become a new channel for citizens to obtain crisis-related information. If the government departments in charge of crisis response fail to deliver official information in a timely manner, citizen information needs may not be satisfied and their channel switching behaviors is unpredictable (7). Consequently, public trust is likely to decline and their willingness, as well as their agreeance to comply with government responses, will decrease. This implies that government communication must leverage social media to increase citizen engagement (8, 9). In addition, social media has empowered citizens with more opportunities to participate in crisis communication, and anyone can now express their opinions on social media. Compared to offline participation, social media-based participation is performed rapidly, including actions such as liking, retweeting, and commenting, which takes minimal effort. In this sense, online participation is more likely to occur and worth extra attention in crisis communication.

Although scholars have begun to place greater emphasis on Citizen Engagement *via* Government Social Media (CEGSM) during public health crises, several limitations are identified. First, extant research has examined government social media accounts to explore the determinants of citizen engagement during public health crises (1, 10–12), while the generality of their conclusions has attracted significant attention. Secondly, the majority of existing literature has explored the influencing factors from a static perspective (5, 13), and overlooked the dynamics of crisis development. However, a recent study into COVID-19 showed that the same factors affect government social media disclosure differently across crisis stages (1). For instance, financial autonomy was observed to positively affect local government information disclosure on Facebook, as measured by the number of tweets across all crises stages, while the role of gender only worked during the pre-warning stage (1). In this sense, it is rational to state that the same factors serve different roles in crisis development when exploring CEGSM. By adding the dynamics of the different crisis stages, the resilience of the research model can be increased, and thus improve the agility of governments to promote citizen engagement. Thirdly, although few efforts have introduced moderators to explain the mechanism of CEGSM (14, 15), the dynamics of crisis stages is also insufficiently considered. In another words, whether mechanisms apply to all crisis stages remains unknown. In addition, despite the efforts on the essence of the content, such as content type (10), extant research has focused extensively on the dialogic loop (14, 15), media richness (16, 17), and emotional valence (12), with few efforts on other important heuristic aspects, including social media capital, language features and their functions. This study, therefore, attempts to adopt the Heuristic-Systematic Model to examine the specific formation mechanisms of citizen engagement with government social media accounts during the different stages of the COVID-19 pandemic. Further, the study fills the above gaps by investigating the differentiated effects of heuristic aspects including social media capital, information richness, language features, dialogic loop, and systematic aspects including content types, on citizen engagement with government social media across the three different stages of the pandemic, including *Swift Response Stage, Initial Containment Stage*, and *Case Drop Stage*, and the moderating role of emotional valence. The proposed model is verified through the scraping of 16,710 posts from 22 provincial and municipal government micro-blog accounts in the Hubei province, China.

## Theoretical Foundations

Dual process theory of human information processing highlights individual decision-making process based on the information provided (18). In their initial setting, human information process is often conceptualized as two different approaches. Among them, the Heuristic-Systematic Model has been widely adopted to explain individual information-related behaviors. For heuristic approach, individuals concentrate on the superficial information clues as demonstrated by the length of the information, the source of the information and how the information is presented. For the systematic approach, individuals care more about the essence of the information as demonstrated in the information content, such as information quality, information usefulness. Prior research has well-documented that these two information processing approaches coincide together when individual making decisions with online information (18–20), for instance women is likely to evaluate the pregnancy-related information obtain through social media by their outlooks and cross-check (21). In terms of the online information reposting behavior, Liu et al. revealed that source trustworthiness, source expertise, source attractiveness and multimedia presentation result in more reposting (18). This is further explained by Shi et al. who found that receiver-related and relationships-related factors are more influential than the source-related factors in attracting reposting (19). In addition, some have suggested that the Heuristic-Systematic Model may apply in crisis communication. For instance, the studies featured on COVID-19 pandemic affirms that message content type, message length and message sentiments contribute to the citizen's online participation behaviors (14, 15). Other variables highlighted are language features, such as first person narratives, and question marks (22).

Building on this theoretical framework, this study looks into how the two information processing approaches affect citizen engage *via* government social media. In our study, the heuristic information mode is manifested as non-central factors, including the social capital of the government social media account, information presentation. And the systematic mode is manifested as central factors, including the content type. By combining the theories of social media capital, dialogic loop and information richness as well as prior research, our research hypotheses concern the social media capital, content type, dialogic loop, information richness, language features, and emotional valence.

## Research Hypotheses

### Content Type and CEGSM

Prior research has demonstrated the different roles of content type on CEGSM. For example, Park et al. revealed that personal health–related information and actions received a greater number of shares and likes by examining the tweets of three American health organizations on Twitter (23). Similarly, Manetti et al. analyzed the content of Facebook posts and Twitter tweets of 35 transportation companies in Canada and the United States and found that content showing entertainment and other auxiliary functions received the most likes (24). In addition, this effect pertains in crises. Meltzer et al. analyzed the Facebook content of 37 emergency management organizations in Romania during crisis situations and found that negative news, rescue stories, achievements of a person or group, or crisis education, attracted most CEGSM (25). Landi et al. analyzed the theme of Facebook posts issued during the COVID-19 epidemic in Italy, the United Kingdom, and New Zealand. Their results showed that the number of likes of posts showing appreciation toward medical staff exceeded the average, and the government action information received a high number of comments (26). Chen et al. studied the government TikTok account of the Healthy China during the COVID-19 crisis, and found that government handling, information, and guidelines, were positive predictors of the number of shares compared to appreciative information (14). Therefore, this study proposes the following hypothesis:

RQ: Will content types affect CEGSM differently?

### Social Media Capital and CEGSM

Social capital has received significant attention by scholars since the early 1980s. It has been widely applied in economics, sociology, politics, knowledge management, information systems, and many other disciplines (27). However, there is no universal definition for social capital. Lin defined social capital as the resources embedded in his own social network, which could be accessed or mobilized by connecting with the network (28). In considering the emerging influence of social media, de Zúiga et al. introduced social capital theory to social media and named it social media social capital, highlighting the role that social media plays in facilitating social capital, and thus distinguishing it from traditional offline social capital in concept and practice (29). Following their efforts, Saxton and Guo proposed a novel resource called social media capital, which refers to the stock of social resources generated by an organization or an individual through its social media efforts (30). To achieve meaningful results through social media activities, organizations must first obtain social media capital. Bourdieu believes that citizen engagement is closely connected to social capital and regards social capital as a collection of real or potential resources, inseparable from having a lasting, recognized, and institutionalized relationship network (31). Similarly, scholars have identified that networks can develop collective identity, building social trust, and providing a new platform for social media capital. For example, Skoric et al. found that online bridging social capital was positively related to online political engagement, and online bonding social capital was positively related to traditional political engagement (32). Other studies have confirmed that when Internet users use social media platforms to obtain information, they promote the formation of social media capital, citizen engagement, and online and offline political engagement (33).

Extant research has indicated a close relationship between social media capital and citizen engagement. The majority of studies have emphasized the role of followers as social capital. For example, Li et al. believe that the number of followers can be conceptualized as social contact. If a social media account has high social contact, it means that the account is popular (34). Shi et al. systematically studied the determinants of personal communication behavior on social media and found that posts by accounts with many followers were more likely to be forwarded by other users (19). Similarly, Liu et al. (18) and Choi et al. (35) confirmed the positive effect of the number of followers on CEGSM. However, Bonson et al. analyzed 22 local government Twitter accounts in Andalusia, Spain, and found that the more followers an account had, the lower the CEGSM (16). In times of crises, social media capital also has an impact on CEGSM. For example, Roy et al. studied tweets related to Hurricane Sandy and found that accounts with many followers attracted higher attention (36). Although research into social media followers has been well-documented, less attention has been given to social media capital, and the role of followees. Putnam divided social capital into bridging social capital and bonding social capital. The bridging relationship between individuals, and social capital, comes from different social backgrounds, in which the bridging relationship between individuals and social capital comes from the different social capital intensity. On the other hand, bonding social capital arises between closely connected individuals and provides emotional and substantive support for individuals (37). For government social media accounts, the number of followers is more connected to bonding social capital, while the number of followees is more connected to bridging social capital. To verify the impact of the social media capital of CEGSM, and their number of followers and followees, we propose the following hypotheses:

H1a. The number of followers has a positive effect on CEGSM.
H1b. The number of followees has a positive effect on CEGSM.

### Dialogic Loop and CEGSM

Kent and Taylor (42) proposed the Dialogic Communication Theory (DCT), which highlights the exchange of ideas and views through negotiation. They emphasized that if organizations want to establish a dynamic and lasting relationship with the public, they must integrate dialogic communication into their network and online communications (38). Correspondingly, they also provided strategies to promote communication with citizens through their website, guiding organizations that intend to establish a two-way dialogic relationship with the public. Specifically, they proposed five principles: Dialogic loop, Usefulness of Information, Generation of Return Visits, Intuitiveness/Ease of the Interface, and Rule of Conservation of Visitors. Based on these five principles, Taylor et al. evaluated the use of the Internet by organizations when establishing relations with the public, and found that compared with protecting visitors, and generating return visits, organizations were more successful in achieving ease of use and information usefulness (39). With the evolvement of media, many scholars have begun to study the use of dialogic principles in social media platforms. For example, Men et al. studied how to implement dialogic principles and social presence strategies in the Facebook posts of senior CEOs to promote citizen engagement. They revealed that the posts had used a variety of dialogic principles and effectively improved citizen engagement (40). Abitbol and Lee summarized three dialogic strategies of disclosure, namely user-friendly message format, and interactivity, and analyzed the situation of 500 enterprises using Facebook's corporate social responsibility page to enhance citizen engagement and confirmed the effect of the dialogic strategies. Their study used the dialogic loop principle to explore the impact of dialogic communication on CEGSM by multiple government agencies in epidemic high-risk areas of the Hubei Province during the COVID-19 pandemic (41).

Dialogic loop is one of the five principles of the DCT, which enables citizens to contact organizations and provide them with the opportunity to answer questions (42). The dialogic loop posits that employees selected within an organization to communicate with the public should master the necessary communication skills, through training, to deal with difficult problems and answer concerns and explain relevant policies. Secondly, the dialogic loop must be complete, that is, to provide a professional and timely response. Communicative responses are a major part of the dialogic loop and are essential for building relationships with citizens. The indicators to measure the level of the online dialogic loop are: (1) User response opportunities; (2) The opportunity to vote on an issue; (3) Survey of opinions on issues; and (4) Provide updated information (38, 39). In regards how dialogic loop affects CEGSM, extant research offers varying conclusions. Wang and Yang studied how non-profit organizations and for-profit organizations use Twitter to establish public dialogic communication. Their results showed that for-profit organizations can increase citizen engagement by emphasizing the principle of dialogic loop (43). In response to the COVID-19 pandemic, Yang et al. (12) studied the official Sina Weibo account of the Wuhan municipal government and found that the use of @ in the dialogic loop had a positive impact on the number of comments and likes received, while the use of labels did not affect them. By analyzing the official TikTok account of the National Health Commission of China during the COVID-19 epidemic, Chen et al. (14) revealed that dialogic loop reduced the number of reposts, but that the number of likes and comments was not affected. Taking single government social media accounts as examples, these two studies offer differing results to that of Wang and Yang (43). To verify the impact of the dialogic loop on multiple Sina Weibo accounts on CEGSM, in the context of public health crises, this study puts forward the following hypothesis:

H2: Dialogic loop positively predicts CEGSM.

### Information Richness and CEGSM

Information richness is a summative term which includes a set of specific clues to reflect whether the information is sufficient or not. Clues to assess information quality include the amount of information, such as the use of pictures, videos, links, writing style, and the number of words (44). With developments in media, information stored on online social media platforms is gradually being presented in richer forms. This information uses pure text, pictures, videos, and hyperlinks, to increase richness (45). Scholars have begun to pay attention to the impact of online information richness on user behavior and are exploring how information richness attracts users' attention (46). During the COVID-19 crisis, government social media accounts have published many posts which have been organized in different forms of simple text or pictures and videos to convey information to citizens to refute rumors and relieve societal anxiety. To evaluate the impact of the different levels of richness on CEGSM in the context of public health crises, building upon the information richness theory, this study classifies the contents of posts published by multiple government social media accounts in the Hubei Province according to the three levels of text, picture, and video, counting the comments received on each post. The number of comments, likes, reposts, and the number of words is calculated to explore whether information richness is one of the factors that affects CEGSM.

There has been extensive study into the relationship between social media-based information and citizen engagement, with many scholars confirming the positive impact of information richness on citizen engagement. For instance, Chu et al. studied the impact of the dominant characteristics of brand posts on consumer engagement and found that pictures, videos, and brand personality characteristics, have a positive impact on the number of comments and reposts received (47). Theiss et al. conducted research related to breast cancer health knowledge posted by the CDC on Facebook and found that citizens prefer to click, repost, comment or praise posts which contain pictures (48). Through comprehensive analysis of posts published by natural disasters management organizations, Liu et al. found that the more words a post contains, the more information it carries, and the more comments and reposting it can attract (17).

However, there is still a dispute regarding the impact of different information presentations on CEGSM, namely text, pictures, and videos. The high richness of information does not always generate greater citizen engagement. Sabate et al. analyzed 164 posts published on the Facebook accounts of five Spanish travel agencies and found that the use of pictures can attract more likes and comments, while videos can increase the number of likes received, but had no impact on the number of comments (49). Yang et al. studied the relationship between the characteristics of information richness published on the official Sina Weibo account of the Wuhan municipal government and CEGSM. Their results showed that the use of links in posts significantly increases the number of comments, likes, and reposts received. Pictures can also increase the number of comments, while videos were the opposite (12). Chen et al. found that the impact of plain text on CEGSM was no less than that of pictures and videos (15). In the case of public health crises, the impact of words, pictures, and videos on CEGSM needs to be further clarified. Therefore, we propose the following hypotheses:

H3a: Media richness positively predicts CEGSM.
H3b: The number of words in a post positively predicts CEGSM.

### Language Features and CEGSM

Language features usually include language content and language style (50). Language content conveys the central idea and meaning of words, while language style reflects the form of communication, which is more closely related to society and psychology (50). In different language styles, interactive language affects citizens' communication intentions and attracts greater engagement (41). The use of personal pronouns and question marks is the key feature of the interactive style. Existing studies find that on social media platforms, first-person pronouns and third-person pronouns can increase group identity and cohesion, while question marks can increase online interaction and trigger greater engagement (41, 50).

With the increasing importance of social media during health crises, scholars have shown significant interest in how language features affect citizen engagement in the context of crisis events. Lee and Yu studied the impact of the language characteristics of Twitter released by the government on CEGSM during the flood in Colorado in 2013. Their results showed that the use of question marks, first-person and third-person pronouns, can effectively promote the number of reposts, in the context of crisis events (22). Stone and Can explored the relationship between language features and CEGSM, and found that third-person pronouns had a positive impact on the number of reposts and likes received (51). Therefore, this study proposes the following hypotheses:

H4a: The use of question marks positively affects CEGSM.
H4b: The use of personal pronouns positively affects CEGSM.

### Emotional Valence as a Moderator

Emotional valence includes positive emotion and negative emotion (52). Existing research has shown that emotional valence has a significant impact on CEGSM. For example, Liu et al. analyzed the Facebook posts released by 55 governments and organizations before and after Hurricane Harvey and found that the use of positive emotions can trigger the engagement behavior of users (17). Similarly, Zavattaro et al. analyzed the posts of GSM accounts and found a positive impact of positive emotion on citizen engagement (53). However, emotional valence is not always positively correlated with citizen engagement. For instance, Stone and Can studied the Twitter accounts of the United States municipal government and found that tweets representing anxiety and anger received a greater number of reposts (51).

Emotional valence may also interact with the specific environment or other factors, which further affects citizen engagement. When a crisis occurs, the environment for information dissemination is full of uncertainty. Citizens use and repost negative emotional information to avoid risks, and the role of negative emotion is more prominent (54). Lee and Yu analyzed Twitter posts related to the 2013 Colorado flood and found that, in an emergency, the use of positive and negative emotions in disastrous posts can have a negative impact on information sharing behavior (22). Chen et al. studied the moderating effect of emotional valence on GSM during the COVID-19 epidemic. They found that posts that demonstrated positive emotions were higher than those containing negative emotions in the posts with high media richness, while specific content types attracted different levels of citizen engagement due to differences in emotional valence (15). Therefore, to verify whether the regulatory effect of emotional valence is still valid for multi-government social media accounts, this study proposes the following hypothesis:

H5: Emotional valence moderates the effects of social media capital, content types, dialogic loop, information richness, and language features, on CEGSM.

During the different stages of a crisis, the emergency response, public mentality, and government measures are also different. The same influencing factors, as well as the moderating role of emotional valence may create different effects across the different stages. Lwin et al. studied how the Health Bureau of Singapore used Facebook to spread information about the Zika epidemic at different crisis stages. Their results showed that different information topics had different values at different crisis stages, and the psychological needs of citizens also changed (55). Yang and Stewart studied Twitter posts during the three stages of Hurricane Harvey, including event preparation, response, and recovery, and found that the posts shared during the different stages had different characteristics, and suggested that government agencies should use different communication strategies during the different stages (56). Therefore, based on the established model, this study will compare the impact of various factors on CEGSM during the different stages of the COVID-19 crisis.

**Figure 1:**
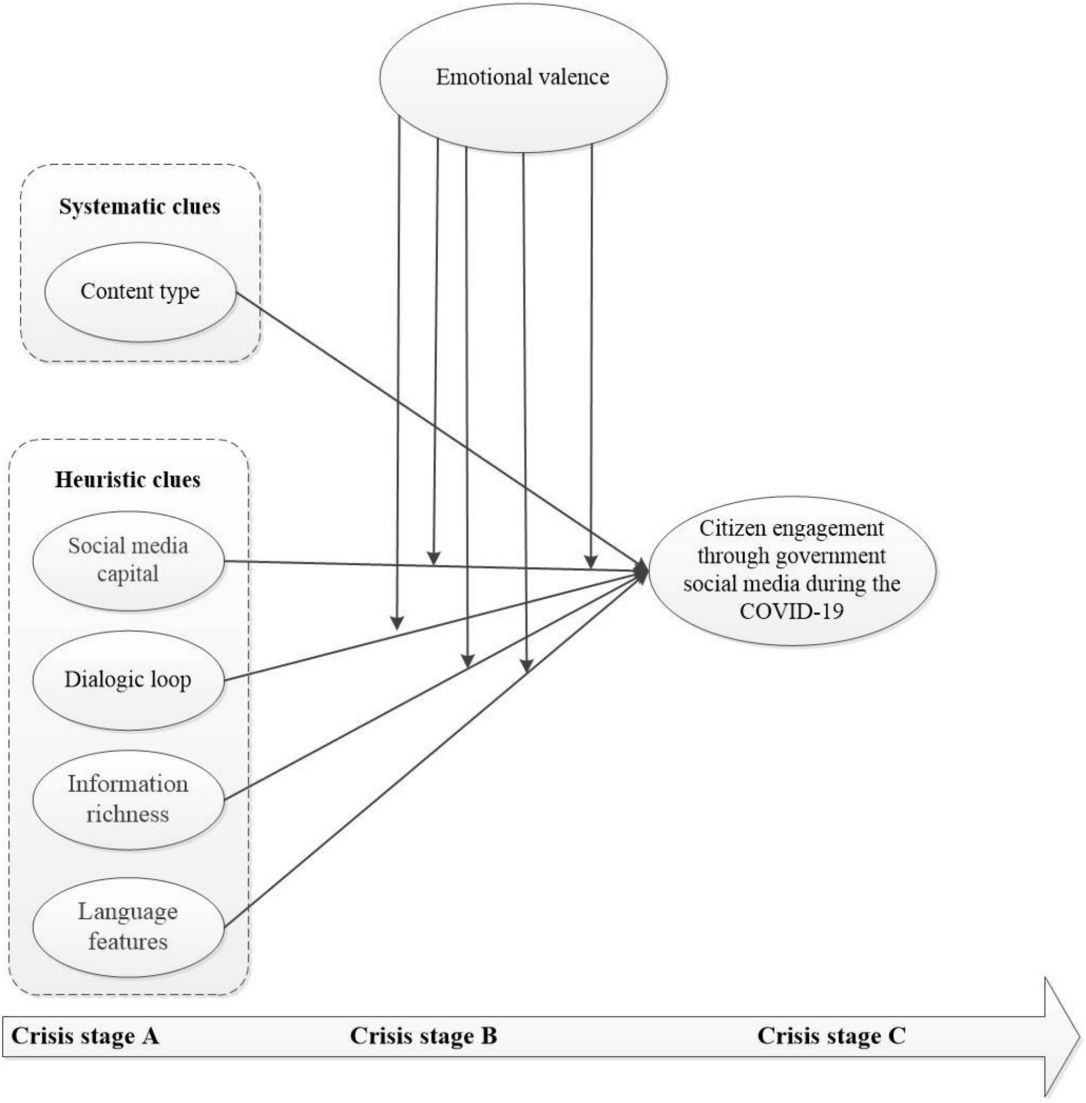
The theoretical model of CEGSM across crisis stages.

To conclude, this study proposes the theoretical model shown in Figure 1.

## Methods

### Data Collection

According to the white paper “*Fighting Covid-19: China in Action,”* published by The State Council Information Office of the People's Republic of China (2020), China's fight against the COVID-19 pandemic can be divided into five stages (57). These stages include: *Stage I: Swift Response to the Public Health Emergency* (27 December, 2019–19 January, 2020), *Stage II: Initial Progress in Containing the Virus* (20 January−20 February, 2020), *Stage III: Newly Confirmed Domestic Cases on the Chinese Mainland Drop to Single Digits* (21 February−17 March, 2020), *Stage IV: Wuhan and Hubei—An Initial Victory in a Critical Battle* (18 March−28 April, 2020), and Stage V: *Ongoing Prevention and Control* (29 April, 2020, onwards). During the period from 27 December, 2019 to 17 March, 2020, the outbreak and spread of COVID-19 caused large-scale panic. The Chinese government took many measures to control the epidemic and citizens relied mainly on social media to learn about the progress of the epidemic. After 17 March, 2020, the local epidemic in Wuhan was deemed under control with control measures for the passage from Wuhan to Hubei being lifted, and the national epidemic prevention and control interdiction war achieving success. This study, therefore, used data from the official Sina Weibo accounts of Chinese governments during Stages I–III (i.e., 27 December, 2019–17 March, 2020) as these are widely considered the most serious stages of the epidemic with the strongest uncertainty for research. For this study, the three stages were renamed as: *Swift Response Stage, Initial Containing Stage*, and *Case Drop Stage*. According to the epidemic risk levels, first released in the Hubei Province on 29 February, 2020, this study selected the Sina Weibo accounts of relevant provincial and municipal government departments in high-risk areas, and removed any accounts that did not release posts during the above period. In total, 22 official accounts were used as data sources. Then, Python was used to capture account information and posts released during the time period. For account information, this study obtained the number of followers and followees. For each post, the text, release time, number of likes, number of comments, number of reposts, and other relevant data, were obtained, while uploaded pictures and / or videos were also captured. A total of 16,710 posts were obtained.

### Operationalization of Variables

CEGSM is composed of the number of likes, reposts, and comments received by each official Sina Weibo account, being measured by the sum of the three components (15).

#### Social Media Capital

For a social media account, the more followers it has, the more bonding social capital it perceives, and the more followees it has, the more bridging social capital it perceives (58). Therefore, this study measures both bonding social media capital and bridging social media capital by the number of followers and followees, respectively, and explores their role against CEGSM.

#### Content Type

Although researchers have studied the content types of posts shared by government social media accounts during the COVID-19 crisis, and provided fundamental frameworks for classification, some content still falls beyond the existing types, for example the content on social donation is frequent, but receives less attention from scholars (15, 26). To fill this gap, this study systematically reviewed and coded the content of posts. The code consists six sub-coders, including 1 for latest news, the number of confirmed epidemic cases at home and abroad, the use of hospital beds and other latest data information; 2 for encouraging information, including appreciation to front-line emergency staff and volunteers, as well as encouraging citizens to adjust their mentality and minimize difficulties together; 3 for guidance information, such as guiding citizens on protective behaviors during the different stages of the epidemic; 4 for government actions, such as documents and policies issued by official government agencies, and the support of the medical team; 5 for rumor refutation, clarifying rumors and punishing rumor mongers; and 6 for social action, including donations and materials donated by social people and enterprises. Table 1 presents the code scheme.

**Table 1:** Content type of posts and example posts.

**Categories**	**Example posts**
Latest news	# Report # [Report from the Wuhan Municipal Health Committee on Pneumonia Infected by COVID-19] From 0:00 to 24:00 on January 12, 2020, there was no new pneumonia cases in our city, and 1 case was cured and discharged, and no new death cases were reported. Up to now, 41 cases of pneumonia infected by COVID-19 have been reported in our city, 7 cases have been cured and discharged, 6 cases are under severe treatment, 1 case has died, and the rest of the patients are in a stable condition. All patients are receiving isolation treatment in designated medical institutions in Wuhan. A total of 763 close contacts have been tracked, 76 people have been released from medical observation, and 687 people are still under medical observation. Among the close contacts, no related cases have been found.
Encouraging information	[Thank you for waiting! # Fully armed soldiers in white #] Pay tribute to all front-line medical staff! Please do your best to protect yourself! United as one, we will surely win this war of epidemic prevention and control! Come on Wuhan #!
Guidance information	[1 minute to understand: # How to protect yourself against COVID-19#?] What is this novel coronavirus? How is it different from previous coronaviruses? How much harm will it cause and how to prevent it? A 1-min video will show you
Government actions	# News Express # [The Ministry of Finance and the NHHC jointly issued a notice to actively implement the funding guarantee policy for epidemic prevention and control] The reporter learned from the Ministry of Finance on the 25th that the Ministry of Finance and the NHHC jointly issued a notice on the funding guarantee policy for pneumonia epidemic prevention and control against the novel coronavirus, actively implemented the funding guarantee policy for epidemic prevention and control, and supported all localities to resolutely curb the spread of the epidemic. The subsidy is coming!
Rumor refutation	# New pneumonia rumors # [Have you seen these 6 rumors?]
Social actions	# Wuhan Anti-epidemic Defense Line # # Wuhan will win # Wuhan Alumni Zhou Feng: Support Wuhan, and all Shanghai alumni will work together.

#### Dialogic Loop

In reference to the five indicators adopted by Chen et al. the dialogic loop is measured by five questions, including: *the use of hashtags, the provision of surveys or votes for users to express opinions, the use of @ function*, r*esponding to a question* and *posting a question*. Each item is coded as either 1 or 0, with 1 being a positive response and 0 being a negative response. The level of dialogic loop is the accumulated value of the five items (15).

#### Information Richness

Information richness includes media richness and the number of words contained in a post. In this study, media richness is divided into three levels, which are plain text (low media richness), pictures or GIFs (medium media richness), and video (high media richness), which are coded as 1, 2, and 3, respectively (15). Further, the number of words contained in each post is calculated by using the Chinese psychoanalysis TextMind software.

#### Language Features

Language features include the use of question marks and personal pronouns. Lee and Yu studied the impact of Twitter post language style on the number of reposts received during the crisis and used inclusive language (first-person pronouns and third-person pronouns) and question marks to measure the interactivity of posts (22). In reference to this method, this study uses the TextMind software to analyze the text of posts and calculates the proportion of question marks and personal pronouns of each post.

#### Emotional Valence

The emotional valence of each post was calculated based on the Sentiment Lexicon with python. Using SnowNLP, this paper divided text into positive and negative categories and calculated the emotional valence for each post. Each post was assigned a weighted emotional valence within the range of 0–1. Zero denoted extreme negative emotion, while 1 denoted extreme positive emotion.

### Data Analysis

Since citizen engagement is count data and the distribution of citizen engagement is over-dispersed (M = 428.62, SD = 22,988.19, skewness = 110.44, kurtosis = 13,078.04), the assumption of normal distribution was violated. To deal with the over-dispersed count data, negative binomial regression is considered appropriate for this study (59). Through negative binomial regression analysis, the impact of social media capital (the number of followers and followees), content type, dialogic loop, information richness (media richness and the number of words), language features (the use of question marks and person pronouns) on CEGSM were studied at the different stages of the epidemic. Then, we explored whether the influence is contingent upon the emotional valence of each post. All analyses were conducted using R version 4.0.4.

## Results

### Descriptive Analysis

The 16,710 posts collected showed significant variation in the level of citizen engagement, with 42.0 percent of posts having <10, while 38 posts were engaged with more than 10,000. The 16,710 posts were divided according to crisis stage. There were 84 posts (0.5%) in the *Swift Response Stage*, 8,735 posts (52.27%) in the *Initial Containment Stage*, and 7,891 posts (47.22%) in the *Case Drop Stage*. There were also noteworthy differences in the number of followers and followees. The number of followers of four accounts exceeded 1 million, while the number of followers of five accounts was <10,000. The account with the most followees followed 916 accounts, while the account with the least followees followed only 4 accounts. Table 2 shows the basic information of the official accounts studied.

**Table 2:** Basic information of the GSM accounts.

**Account name**	**Administrative level**	**Government department**	**Registration time**	**Number of followers**	**Number of followees**	**Number of posts**
Emergency management department of Hubei province	Provincial	Emergency	2015-3-16	10,934	162	99
Hubei publish	Provincial	News	2011-12-26	1,896,264	256	1,440
Hubei provincial government portal website	Provincial	Local	2012-6-15	1,592,815	237	1,302
Healthy Wuhan official Weibo	Municipal	Health	2019-12-31	54,996	4	346
Wuhan disease control	Municipal	Health	2021-1-25	468	15	27
Wuhan emergency management	Municipal	Emergency	2012-5-17	3,138	116	90
Wuhan publish	Municipal	News	2013-7-9	3,787,698	498	3,525
Ezhou publish	Municipal	News	2011-7-26	128,446	40	540
Ezhou government network	Municipal	Local	2013-12-27	43,629	134	648
Huanggang government portal	Municipal	Local	2014-9-26	25,062	282	560
Huangshi publish	Municipal	News	2015-7-17	62,890	203	1,860
Jingzhou publish	Municipal	News	2015-1-16	151,296	493	668
Jingzhou emergency management	Municipal	Emergency	2012-3-5	3,182	27	18
Shiyan publish	Municipal	News	2014-3-26	124,133	299	91
Charming Shiyan	Municipal	News	2011-3-16	135,717	160	273
Suizhou municipal government portal	Municipal	Local	2015-11-3	5,692	80	736
Xianning publish	Municipal	News	2013-5-15	13,006	174	664
Xiangyang publish	Municipal	News	2012-2-28	51,018	57	363
China Xiangyang government network	Municipal	Local	2011-11-2	57,035	72	373
Xiangyang emergency management	Municipal	Emergency	2012-4-30	2,777	67	49
Yichang publish	Municipal	Local	2011-3-13	961,514	916	1,343
Xiaogan publish	Municipal	News	2012-10-16	1,181,810	315	1,695

**Figure 2:**
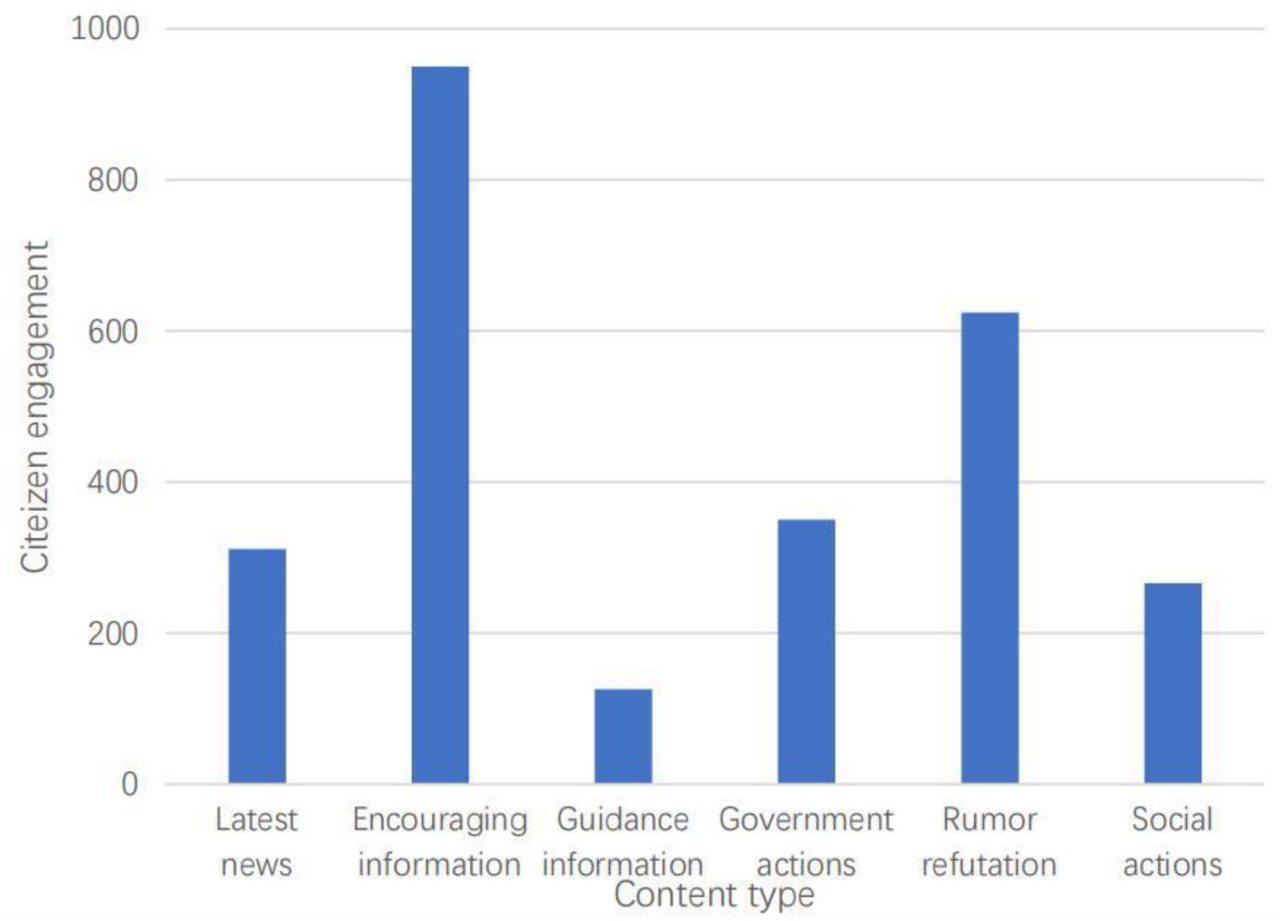
Average volume of citizen engagement grouped by content type.

Among all the posts, 7,338 (43.9%) were related to government actions, followed by encouraging information (21.0%, *n* = 3,510), guidance information (14.9%, *n* = 2,484), social actions (8.0%, *n* = 1,333), and the latest news (10.6%, *n* = 1,769). There were only 276 posts relating to rumor refutation (1.6%). On average, posts that shared encouraging information received the highest citizen engagement (M = 950.19, SD = 47,091.22), followed by rumor refutation (M = 624.54, SD = 6,806.02), social actions (M = 166.42, SD = 1,165.28), and guidance information (M = 126.11, SD = 585.04) (see Figure 2 for details). Of the dialogic features analyzed, hashtags (61.0%, *n* = 10,185) were the most used tool, followed by @ (8.4%, *n* = 1,407), and answering questions (7.1%, *n* = 1,179). Almost no questions, surveys or votes were published. In terms of media richness, nearly 58.1% of posts (*n* = 9,712) used pictures to spread information, 6,130 posts used plain text, and 868 posts used videos (5.2%, *n* = 868). There was also a sharp difference in the number of words contained in posts. There were 8,990 posts with <140 words (53.8%), while there were only 278 posts with more than 1,000 words (1.66%). Few posts used question marks (4.05%, *n* = 676), while 33.6% used first-person and third-person pronouns to enhance the interaction of posts (*n* = 5,622). Furthermore, the emotional valence of the posts was positive, on average (M = 0.56, SD = 0.46).

### Hypothesis Tests

Table 3 shows the results of our negative binomial regression model predicting CEGSM during the COVID-19 crisis. Model 1 and 2 presents the results of the *Swift Response Stage*. Models 3 and 4 present the results of the *Initial Containing Stage*. Models 5 and 6 present the results of the *Case Drop Stage*. Models 1, 3, and 5 are models that predict the main effect, while models 2, 4, and 6 introduce emotional valence as the moderating variables to predict the interaction effect.

**Table 3:** Predicting CEGSM in the three stages of the COVID-19 crisis.

	**Model 1**		**Model 2**		**Model 3**		**Model 4**		**Model 5**		**Model 6**	
	**IRR**	**SE**	**IRR**	**SE**	**IRR**	**SE**	**IRR**	**SE**	**IRR**	**SE**	**IRR**	**SE**
(Intercept)	1.15	0.63	3.07	1.91	1.66	0.05***	2.01	0.08***	1.31	0.06***	1.54	0.09***
**Main effect**												
**Social media capital**												
Number of followers	1.00	0.00***	1.00	0.00*	1.00	0.00***	1.00	0.00***	1.00	0.00***	1.00	0.00***
Number of followees	1.00	0.00**	1.00	0.00	1.00	0.00***	1.00	0.00***	1.00	0.00***	1.00	0.00***
**Content type (reference group: latest news)**												
Encouraging information	NA	NA	NA	NA	0.84	0.02***	0.85	0.04***	0.70	0.02***	0.72	0.03***
Guidance information	1.01	0.19	0.94	0.33	0.78	0.03***	0.83	0.04***	0.74	0.03***	0.81	0.04***
Government actions	0.85	0.16	0.81	0.38	0.78	0.02***	0.80	0.03***	0.65	0.02***	0.70	0.03***
Rumor refutation	0.70	0.37	0.95	0.49	0.86	0.05***	0.83	0.07**	0.73	0.06***	0.80	0.08**
Social actions	NA	NA	NA	NA	0.95	0.03	0.94	0.04	0.74	0.03***	0.75	0.04***
Dialogic loop	1.08	0.11	1.02	0.33	1.07	0.01***	1.07	0.02***	1.03	0.01*	1.00	0.02
**Information richness**												
Media richness	1.08	0.01	0.84	0.32	1.07	0.01***	1.06	0.02***	1.08	0.01***	1.05	0.02*
Number of words	1.11	0.01	0.95	0.41	1.02	0.01**	1.00	0.01	1.07	0.01***	1.09	0.01***
**Language features**												
Question marks	1.05	0.07	0.91	0.15	0.97	0.01**	0.98	0.01	0.97	0.01**	0.96	0.02
Person pronouns	1.04	0.07	1.24	0.17	0.99	0.01	0.99	0.01	0.99	0.01	0.99	0.01
**Interaction effect**												
Emotional valence			0.37	2.20			0.69	0.12**			0.67	0.13**
EV*Number of followers			1.00	0.00			1.00	0.00***			1.00	0.00*
EV*Number of followees			1.00	0.00			1.00	0.00***			1.00	0.00***
EV*Content type (reference group: latest news)												
Encouraging information							1.00	0.05			1.07	0.05
Guidance information			1.10	0.46			0.91	0.06			0.91	0.06
Government actions			1.08	0.44			0.97	0.05			0.94	0.05
Rumor refutation			0.19	1.60			1.08	0.10			0.88	0.14
Social actions							1.04	0.06			1.07	0.07
EV*Dialogic loop			1.07	0.37			1.00	0.02			1.04	0.03
EV*Information richness												
EV*Media richness			1.49	0.38			1.03	0.02			1.06	0.03*
EV*Number of words			1.15	0.46			1.05	0.02*			1.00	0.02
EV*language features												
EV*Question marks			1.28	0.22			0.99	0.02			1.02	0.03
EV*Personal pronouns			0.80	0.20			1.00	0.02			0.99	0.02
Log likelihood		−143.24		−139.53	−15,840.49	−15,821.98		−14,002.15		−13,933.39
Pseudo *R^2^* (%)		17.99		20.11		9.82		9.93		10.97		11.41
*N*		84		84		8,735		8,735		7,891		7,891

IRR, Incident Rate Ratio; SE, Standard Error; EV, Emotional Valence; *p < 0.05; **p < 0.01; ***p < 0.001.

RQ1 argued that CEGSM depended on the content type of posts. The content type was classification variable and latest news was regarded as a reference group. The results of model 1 showed that in the *Swift Response Stage*, content type involved only latest news, guidance information, government actions, and rumor refutation, and the impact of content type on CEGSM was not significant. The results of model 3 showed that during the *Initial Containment Stage*, encouraging information (IRR = 0.84, *P* < 0.001), guidance information (IRR = 0.78, *P* < 0.001), government actions (IRR = 0.78, *P* < 0.001), and rumor refutation (IRR = 0.86, *P* < 0.001), had a negative impact on CEGSM. This means that, compared with the latest news, encouraging information reduces the level of CEGSM by 16%, while guidance information reduces the level of CEGSM by 22%, government actions would reduce the level of CEGSM by 22%, rumor refutation would reduce the level of CEGSM by 14%, and the effect of social actions on CEGSM was not obvious, compared with government actions. The results of model 5 showed that in the *Case Drop Stage*, encouraging information (IRR = 0.70, *P* < 0.001), guidance information (IRR = 0.74, *P* < 0.001), government actions (IRR = 0.65, *P* < 0.001), rumor refutation (IRR = 0.73, *P* < 0.001), and social actions (IRR = 0.74, *P* < 0.05), had a negative impact on CEGSM. This meant that, compared with latest news, encouraging information would reduce the level of CEGSM by 30%, guidance information would reduce the level of CEGSM by 26%, government actions would reduce the level of CEGSM by 35%, rumor refutation would reduce the level of CEGSM by 27%, and social actions would reduce the level of CEGSM by 26%. Therefore, the content type partially affects CEGSM differently.

H1a proposed that the greater the number of followers to an account, the higher the level of CEGSM. The results of model 1 showed that during the *Swift Response Stage*, the number of followers [Incident Rate Ratio (IRR) = 1.00, *P* < 0.001] had a positive impact on CEGSM. The results of model 3 showed that during the *Initial Containment Stage*, the number of followers (IRR = 1.00, *P* < 0.001) had a positive impact on CEGSM. The results of model 5 showed that during the *Case Drop Stage*, the number of followers (IRR = 1.00, *P* < 0.001) had a positive impact on CEGSM. Therefore, H1a was supported. H1b proposed that the number of followees can improve the level of CEGSM. The results of model 1 showed that during the *Swift Response Stage*, the number of followees (IRR = 1.00, *P* < 0.01) had a positive impact on CEGSM. The results of model 3 showed that during the *Initial Containment Stage*, the number of followees (IRR = 1.00, *P* < 0.001) had a positive impact on CEGSM. Finally, the results of model 5 showed that in the *Case Drop Stage*, the number of followees (IRR = 1.00, *P* < 0.001) had a positive impact on CEGSM. Therefore, H1b was supported.

H2 argued that dialogic loops, such as the use of hashtags, @, and raising or answering questions, could increase CEGSM. The results of model 1 showed that the role of dialogic loop was not significant in the *Swift Response Stage."* The results of model 3 showed that during the *Initial Containment Stage*, dialogic loop (IRR = 1.07, *P* < 0.001) had a positive impact on CEGSM. An increase of one unit in the level of dialogic loop would increase the level of CEGSM by 7%. The results of model 5 showed that during the *Case Drop Stage*, the dialogic loop (IRR = 1.03, *P* < 0.05) had a positive impact on CEGSM. An increase of one unit in the dialogic loop level would increase the level of CEGSM by 3%. Therefore, H2 was partially supported.

H3a proposed that information richness would increase CEGSM. The results of model 1 showed that the role of information richness was not significant in the *Swift Response Stage*. The results of model 3 showed that during the *Initial Containment Stage*, there was a significant positive correlation between media richness (IRR = 1.07, P < 0.001) and CEGSM. An increase of one unit of media richness would increase the level of CEGSM by 7%. The results of model 5 showed that there was a significant positive correlation between media richness (IRR = 1.08, P < 0.001) and citizen CEGSM in the *Case Drop Stage*. An increase of one unit of media richness would increase the level of CEGSM by 8%. Therefore, H3a was partially supported. H3b argued that the greater the number of words contained in a post, the higher the level of CEGSM. The results of model 1 showed that the number of words was not a key effect on CEGSM in the *Swift Response Stage*. The results of model 3 showed that during the *Initial Containment Stage*, the number of words (IRR = 1.02, *P* < 0.01) had a positive impact on CEGSM. For each unit of words, the level of CEGSM increases by 2%. The results of model 5 showed that in the *Case Drop Stage*, the number of words (IRR = 1.07, *P* < 0.001) had a positive impact on CEGSM. For each unit of words, the level of CEGSM increased by 7%. Therefore, H3b was partially supported.

H4a proposed that the use of question marks would increase the level of CEGSM. The results of model 1 showed that the role of question marks was not significant in the *Swift Response Stage*. The results of model 3 showed that in the *Initial Containment Stage*, the use of question marks (IRR = 0.97, *P* < 0.01) had a negative impact on CEGSM. Increasing the use of question marks by one unit would reduce the level of CEGSM by 3%. The results of model 5 showed that during the *Case Drop Stage*, the use of question marks (IRR = 0.97, *P* < 0.01) had a negative impact on CEGSM. Increasing question marks by one unit would reduce the level of CEGSM by 3%. Therefore, H4a was not supported. H4b argued that the use of personal pronouns would positively affect CEGSM. The results of models 1, 3, and 5 showed that the role of person pronouns was not significant across the three stages. Therefore, H4b was not supported.

**Figure 3:**
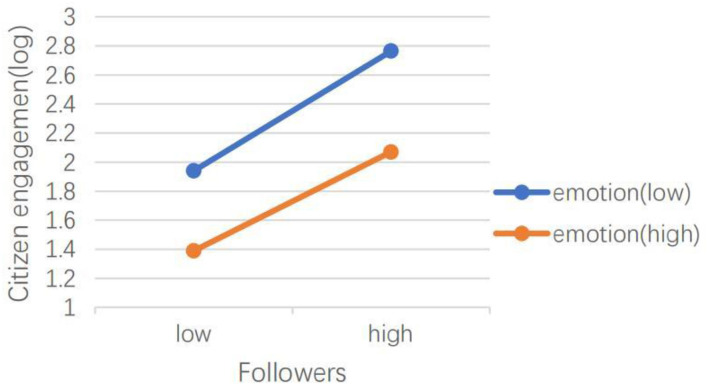
Two-way interaction between the number of followers and emotional valence in predicting CEGSM in the initial containment stage.

**Figure 4:**
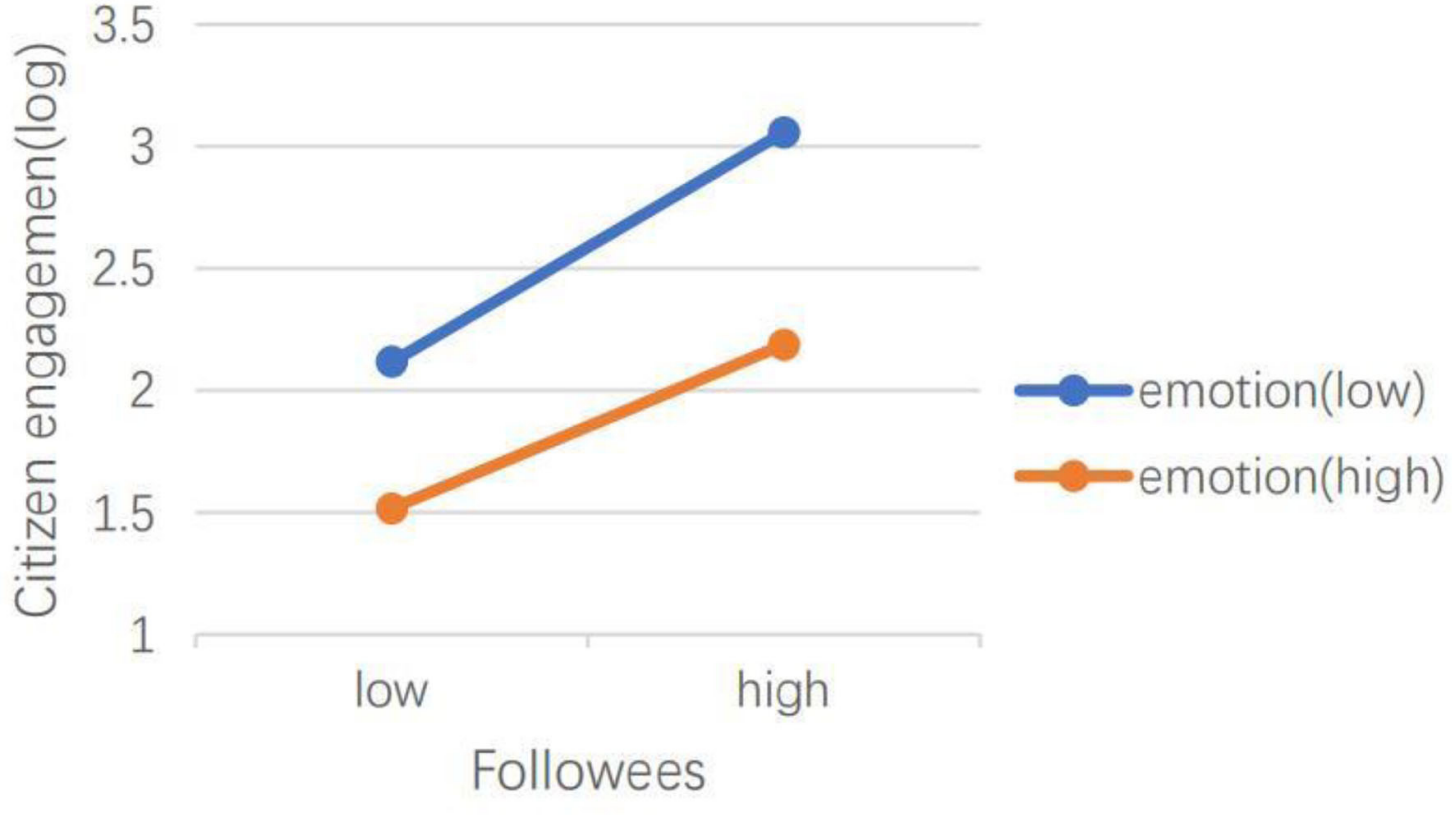
Two-way interaction between the number of followees and emotional valence in predicting CEGSM in the initial containment stage.

**Figure 5:**
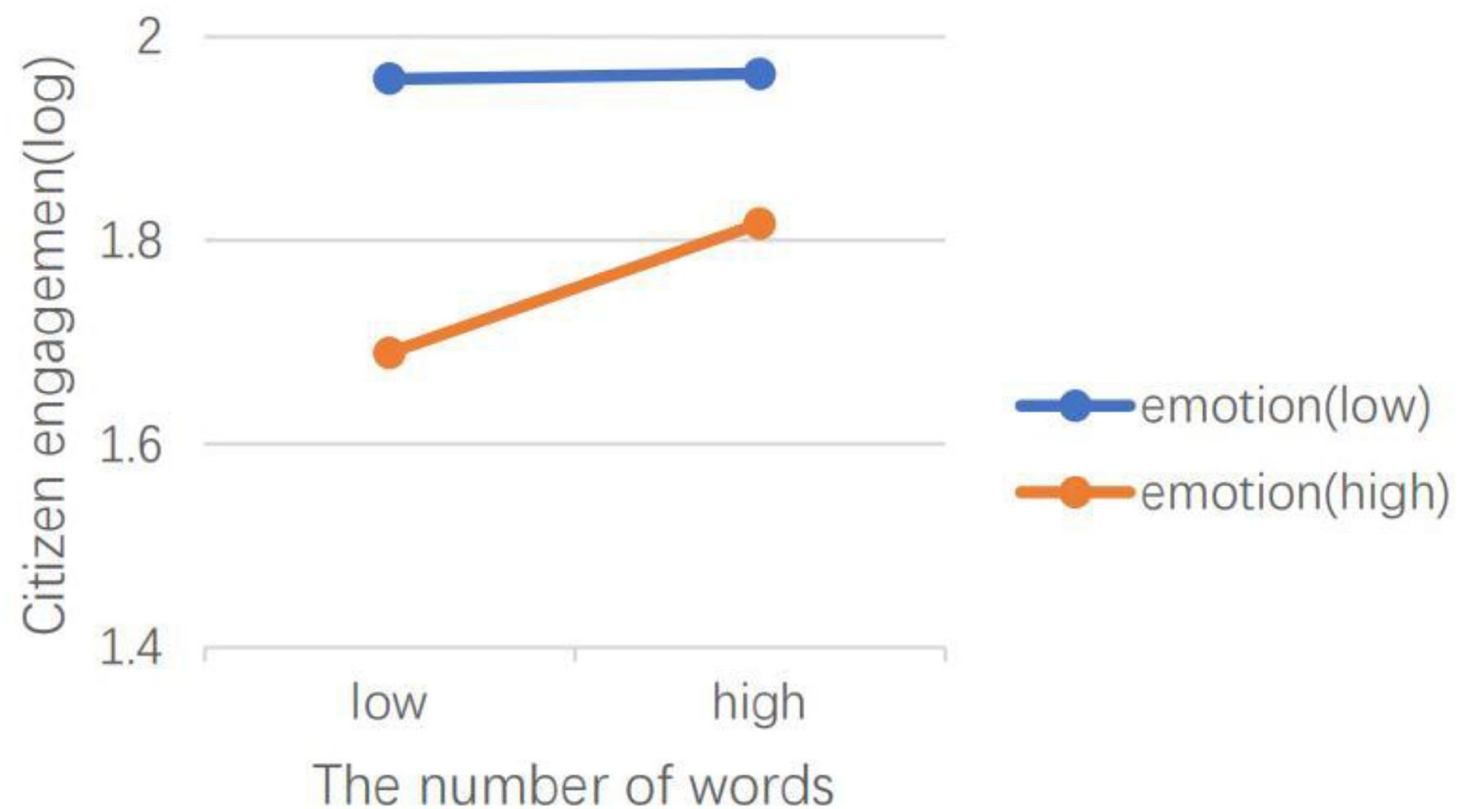
Two-way interaction between the number of words contained in posts and emotional valence in predicting CEGSM in the initial containment stage.

**Figure 6:**
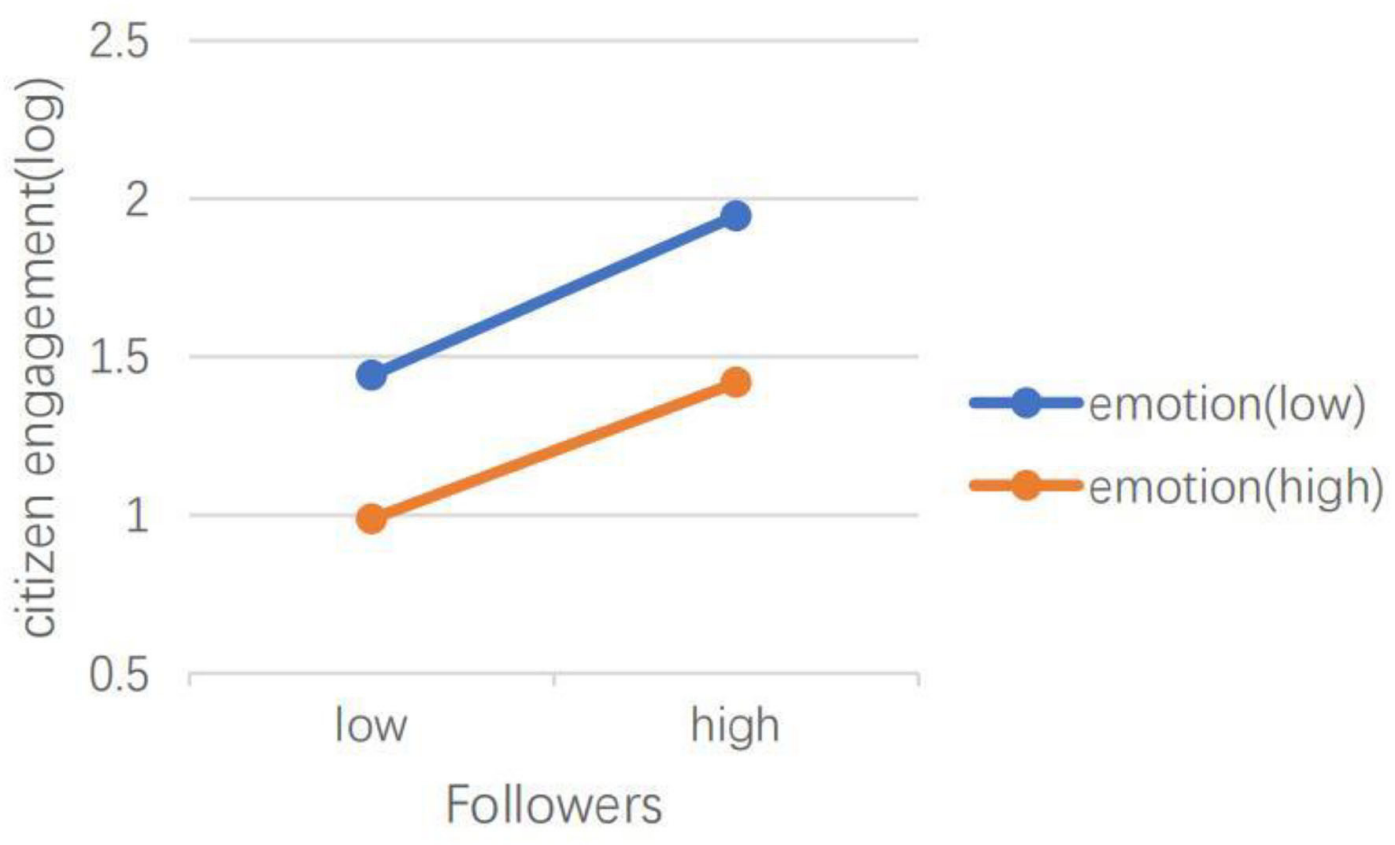
Two-way interaction between the number of followers and emotional valence in predicting CEGSM in the case drop stage.

**Figure 7:**
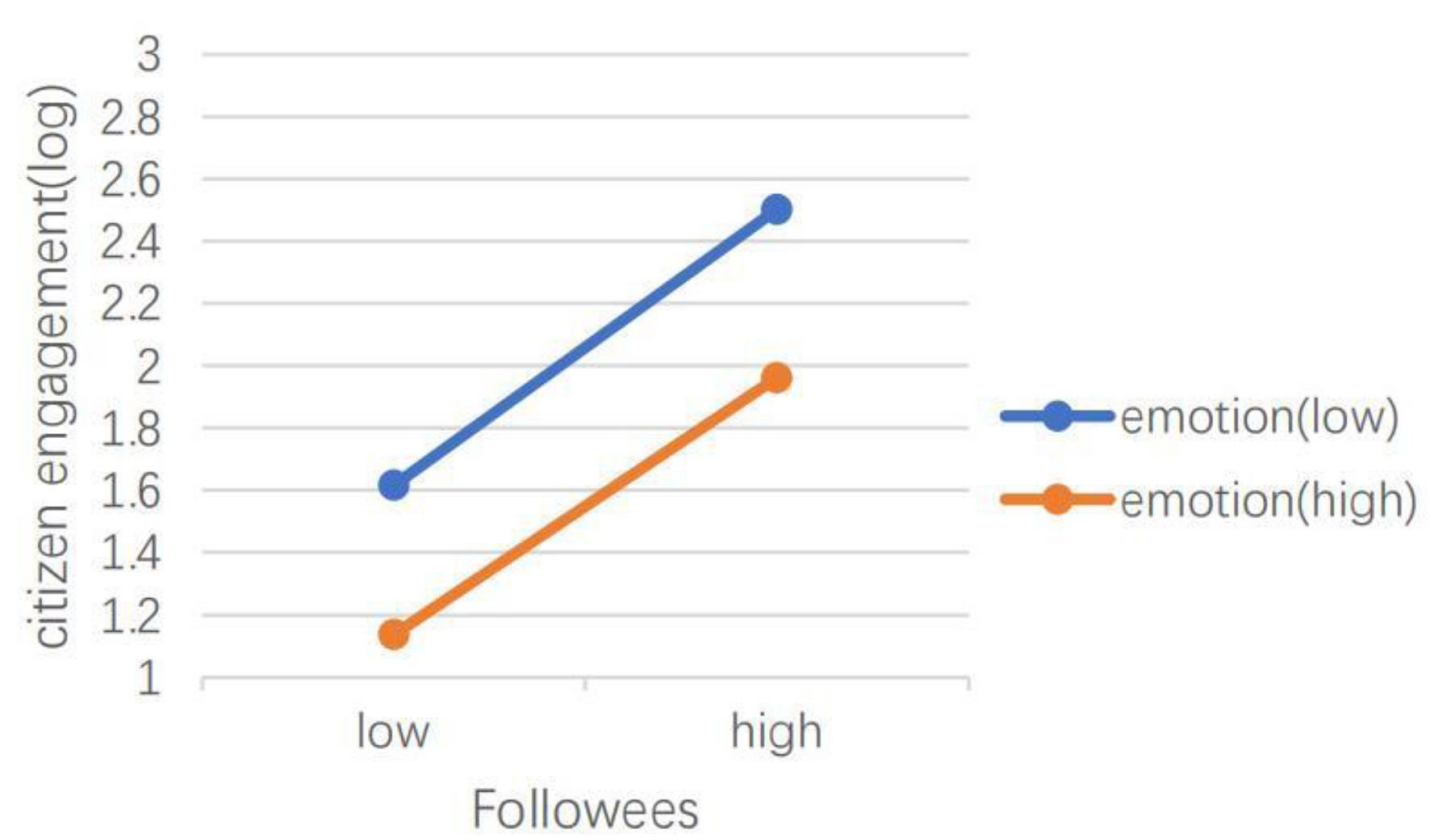
Two-way interaction between the number of followees and emotional valence in predicting CEGSM in the case drop stage.

**Figure 8:**
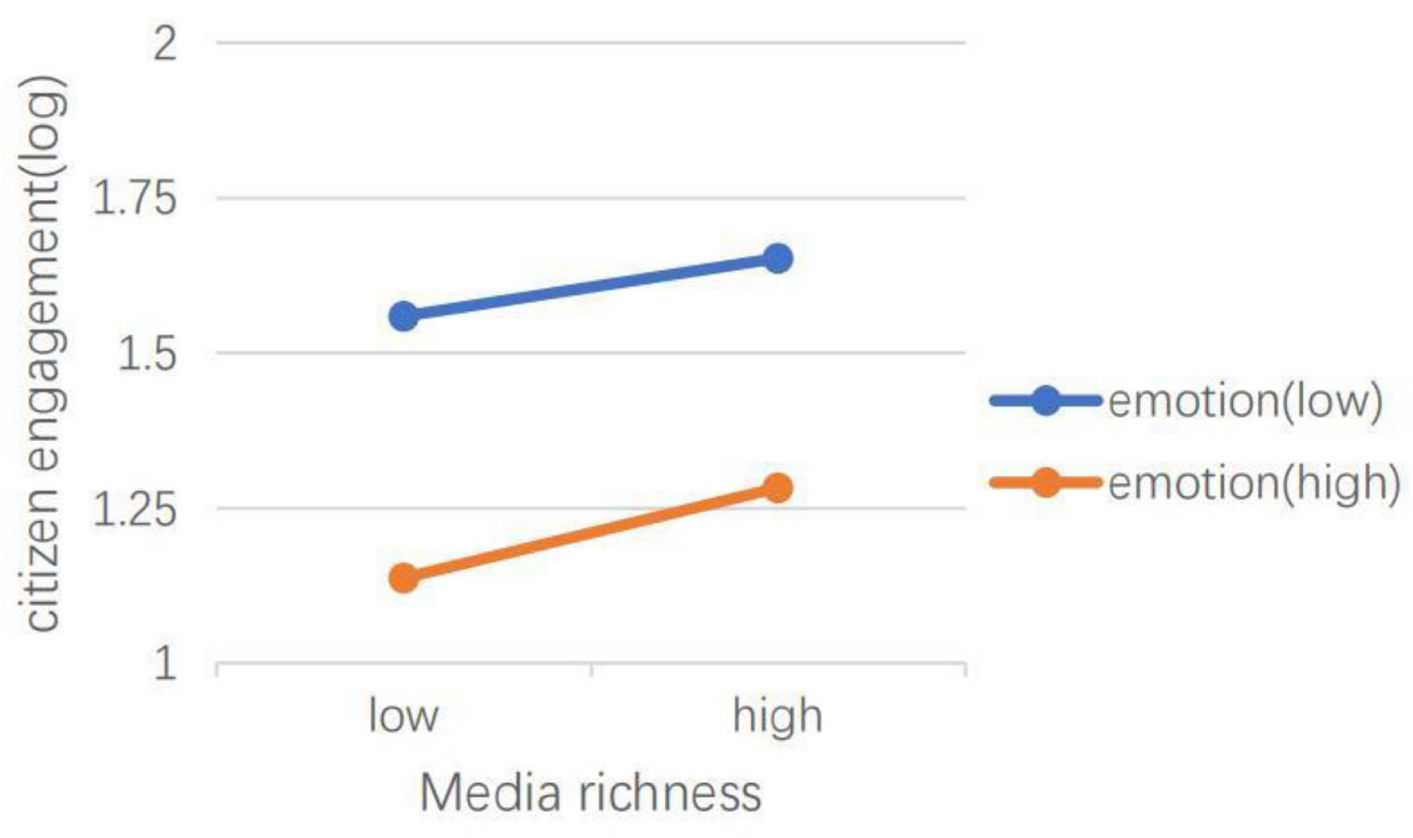
Two-way interaction between media richness and emotional valence in predicting CEGSM in the case drop stage.

H5 hypothesized that emotional valence can moderate the effects of social media capital, content type, dialogic loop, information richness, and language features of social media on CEGSM. The results of model 2 showed that in the *Swift Response Stage*, the moderating effect of emotional valence was not significant. The results of model 4 showed that during the *Initial Containment Stage*, emotional valence moderated the relationship between the number of followers (IRR = 1.00, *P* < 0.001), the number of followees (IRR = 1.00, *P* < 0.001), and the number of words (IRR = 1.05, *P* < 0.05), and CEGSM. As shown in Figures 3, 4, the difference between posts containing positive emotions and negative emotions is more significant when there are many followers and followees. As shown in Figure 5, the difference between positive emotion posts and negative emotion posts is more significant when the number of words contained in posts is small. The more negative emotions displayed in a post, the higher the level of CEGSM. The results of model 6 showed that in the *Case Drop Stage*, the emotional valence moderated the relationship between the number of followers (IRR = 1.00, *P* < 0.05), the number of followees (IRR = 1.00, *P* < 0.001), media richness (IRR = 1.06, *P* < 0.05), and CEGSM. As shown in Figures 6, 7, the difference between positive emotion posts and negative emotion posts is most significant when there are many followers and followees. As shown in Figure 8, the difference between posts with positive emotions and negative emotions is more significant when the media richness degree is low. The more negative emotions displayed in a post, the higher the level of CEGSM. In conclusion, H5 was partially supported.

## Discussion

### Summary of Findings

This paper discusses how official GSM accounts in the Hubei Province affected CEGSM during the COVID-19 crisis. To achieve this goal, based on Social Media Capital Theory, Dialogic Communication Theory and Information Richness Theory, this study systematically studied the influence of social media capital, content type, dialogic loop, information richness, and language features, on CEGSM. More importantly, this study introduced emotional valence as a moderating variable to reveal the potential mechanisms of CEGSM and compared the differences of influencing factors of CEGSM at the different stages of the COVID-19 crisis.

During the *Swift Response Stage*, social media capital played a positive role in CEGSM. Consistent with the conclusions of existing studies, the more followers and followees an account possesses, the higher the level of CEGSM. The more followers that an official government social media account has, the more pay attention and browse more frequently, while the number of reposts, likes, and comments also increases (60, 61). Other influencing factors did not play a significant role in the *Swift Response Stage*. The possible reason for this is that in the early days of the outbreak, citizens lacked awareness of the epidemic and did not have a strong sense of crisis. CEGSM was only related to social media, such as followers and followees. On the other hand, during the early stage of the crisis, the information supply of government agencies about the epidemic situation was insufficient, and many government social media accounts were still publishing other content unrelated to the epidemic situation. This flooded the information related to the COVID-19 epidemic, resulting in citizens' failure to understand the crisis situation in a timely and profound manner, decreasing the CEGSM.

In the *Initial Containment Stage*, firstly, compared with latest news, encouraging information, guidance information, government actions, and rumor refutation, was observed to reduce CEGSM. Different content types can lead to different CEGSM, which is consistent with the conclusions of previous studies (24–26). As for social media capital, it had a positive effect on CEGSM, which was consistent with the conclusions of extant research. The dialogic loop would increase the level of CEGSM, which is consistent with previous findings (43). According to the Dialogic Communication Theory, if an organization wants to establish a relationship with citizens, it needs to incorporate dialogic communication into online communications (42). In terms of information richness, the media richness and number of words contained in posts had a positive impact on CEGSM. The higher the media richness and the number of words, the greater the number of received reposts, likes, and comments, which is in line with the conclusions of existing studies (16, 62, 63). Hofmann et al. found that pictures and videos contribute to the success of online communication when studying the factors that promote the government's successful communication on social media (62). In addition to media richness, the positive impact of the number of words on CEGSM was also consistent with the conclusion that the number of words can promote CEGSM in existing literature (12, 17). In terms of language features, the use of question marks reduces CEGSM, while the role of personal pronouns was not significant. This conclusion was different from the findings of existing studies. Lee and Yu found that the use of personal pronouns and question marks had a positive impact on CEGSM (22). The results of Stone and Can showed that the number of third-person pronouns was positively correlated with the number of reposts and likes (51). After the introduction of emotional valence as a moderating variable, the number of followers and followees of accounts could still increase CEGSM. The role of content type, dialogic loop, media richness, and language features, on CEGSM was no longer significant. Posts that contained a large number of words and negative emotions can improve the level of CEGSM.

During the *Case Drop Stage*, compared with latest news, posts containing encouraging information, guidance information, government actions, rumor refutation, and social actions, would reduce CEGSM, which verified the different effects of different content types on CEGSM. For social media capital, it could improve the level of CEGSM. We found that government social media accounts with more followers and followees obtained more CEGSM, which is consistent with the conclusions of existing studies. The dialogic loop would increase the level of CEGSM, which was consistent with existing research conclusions (43). In terms of information richness, media richness can increase the number of reposts, likes, and comments received. This conclusion is the same as existing research. For example, Bonson et al. studied the impact of the media types of tweets published by government social media accounts. Compared to other media types, pictures, and videos can quickly and intuitively transmit information, so as to improve the number of collections and reposts (16). Lappas et al. studied Facebook accounts in five cities in Greece and found that posts containing pictures, videos, and links, received more likes than pure text content (63). This study also verified the conclusion that the number of words in the existing research would increase CEGSM (12, 17). In terms of language features, as in the *Initial Containment Stage*, the use of question marks would reduce CEGSM, and the role of personal pronouns was not significant. After the introduction of emotional valence as a moderating variable, the number of followers and followees of accounts still had a positive effect on CEGSM. The effect of content type, dialogic loop, the number of words, and language features, on CEGSM was no longer significant. Posts with high media richness and negative emotions attract more CEGSM.

### Comparison of CEGSM in the Three Different Crisis Stages

This study confirmed that the influencing factors of CEGSM are different during the different stages of the crisis. First, the positive effect of the number of followers and followees on CEGSM ran through the *Swift Response Stage, Initial Containment Stage* and *Case Drop Stage*. Sina Weibo accounts with a large number of followers and followees meant more social capital and stronger social ties, which is more likely to be widely spread (34). Second, content type played different roles during the different stages. In the *Swift Response Stage*, due to the outbreak of the epidemic, there were few epidemic-related contents released by government social media, and the types only involved latest news, guidance information, government actions, and rumor refutation. At this time, the CEGSM was low and was not affected by the content type. In the following two stages, compared with latest news, posts containing encouraging information, guidance information, government actions, and rumor refutation, would influence CEGSM. The role of social actions was not significant in the *Initial Containment Stage* but was significant in the *Case Drop Stage*. During the entirety of the COVID-19 crisis, citizens paid most attention to the latest news to reduce anxiety and uncertainty, and to understand the latest developments of the epidemic situation.

Third, the dialogic loop promoted CEGSM in the *Initial Containment Stage* and the *Case Drop Stage*. Through the investigation of posts, this study found that during the *Swift Response Stage*, government social media accounts had not formed a fixed mode of publishing information, and published information was relatively simple. Since the *Initial Containment Stage*, various government social media accounts used many hashtags in posts and published and answered many questions. Meanwhile, many posts used the way of self-questioning and self-answering to establish a two-way dialogic relationship with the public, providing communication channels, actively feeding back citizens' problems, and effectively improving the level of dialogic loop and attracting public attention. Fourth, information richness played a positive role in the *Initial Containment Stage* and the *Case Drop Stage*. Media richness was positively correlated with CEGSM, and the number of words was observed to increase CEGSM. When public health emergencies occur, citizens tend to comment and repost posts with rich forms and containing many words. The use of pictures, videos, and a large number of words, can improve the richness of information and improve the level of CEGSM. Fifth, in terms of language features, the use of question marks had a negative impact on CEGSM, while personal pronouns had no significant effect. The possible reason for this is that in public health emergencies, personal pronouns and question marks may increase public anxiety and reduce the level of CEGSM.

Sixth, emotional valence played a moderating role in the *Initial Containment Stage* and the *Case Drop Stage*. Before the introduction of emotional valence as a moderating variable, the dialogic loop, media richness, and the number of words in the *Initial Containment Stage* and the *Case Drop Stage* had a positive effect on CEGSM, and the question marks had a negative effect on CEGSM. After introducing emotional valence as a moderating variable, emotional valence moderated the impact of information richness on CEGSM. During the *Initial Containment Stage*, posts containing a large number of words and negative emotions could improve CEGSM, while in the *Case Drop Stage*, posts with high media richness and containing negative emotions could improve the level of CEGSM. To sum up, the characteristics of each stage of the crisis are different, and the effects of various factors on the CEGSM also vary. Government agencies should, therefore, formulate corresponding crisis communication strategies according to the different development stages of the crisis, to accurately meet the information needs of citizens.

### Theoretical Implications

Our study contributes to extant research on CEGSM during crisis by integrated the theories of Heuristic-Systematic Model and crisis stages. First, our study further revealed that social media capital facilitates CEGSM during the entire crisis, which is supplement to the current knowledge regarding on how social media capital affects citizen engagement in different crisis stages. Second, considering the crisis stages, our study enriches prior conclusion about how content type affects CEGSM, and found their differentiations across crisis stages. In addition, our study has systematically examined the role of dialogic loop, information richness, language features, as well as the moderating role of emotional valence, and identified several differentiations between crisis stages. This will contribute to a more dynamic understanding of these variables in affecting CEGSM, and thus complete extant theoretical framework.

### Practical Implications

The findings provide implications for the further effective use of GSM, which is beneficial to the government's efforts to improve health communication strategies, and better cope with crises.

First, governments should leverage the use of social media capital. Citizens pay greater attention to government social media accounts that possess large numbers of followers and followees. Governments must, therefore, strengthen the operation of government social media accounts in daily situations, publish content to attract citizens, increase the number of followers and followees, strengthen the social relations of accounts, and obtain the dependence and trust of the public. In the event of a crisis, governments should use their official accounts to convey information to increase CEGSM. Government agencies should arrange full-time managers to manage their accounts in order to optimize government social media and attract citizen engagement as well.

Secondly, in addition to making rational use of social capital, government agencies must pay attention to the content and form of information shared. During the COVID-19 epidemic, the posts shared by government social media accounts were lively and vivid, concise, and easy to understand with a lot of charts, diagrams, audio and videos, etc. To release important policies and information that the public pays greater attention to, and to provide timely feedback, governments must continue to do this. In terms of the content and form of information, we suggest the following: in terms of content, governments should use technical means to analyze the information needs of citizens at different stages of public health emergencies, understand the content most concerned by citizens, and formulate the content type of posts, according to the results of analysis. After citizens' needs are met, they will have more trust in the GSM account, which is conducive to promoting CEGSM in daily or emergency events, in the future. In terms of form of information, government agencies should make full use of dialogic loop, media richness, and the number of words, to enhance CEGSM. When a public health crisis occurs, in order to eliminate anxiety and uncertainty, citizens urgently require more authoritative information from government agencies. Government agencies can add hashtags or @ in posts, publish questions, answer questions, provide surveys and votes, etc. to improve the level of dialogic loop, and use pictures and videos in posts to enhance the richness of media, and increase the number of words in posts, so as to promote CEGSM. In addition, the use of question marks and personal pronouns may reduce CEGSM. However, it is necessary to control the proportion of question marks and personal pronouns in the post.

In addition, when disseminating information and communicating online, government agencies must consider the moderating effect of emotional valence on other influencing factors. The results of this study show that emotional valence could adjust the impact of the number of followers and followees of government social media accounts on CEGSM, while negative emotions can adjust the impact of the number of words on CEGSM in the *Initial Containment Stage*, and the impact of media richness on CEGSM in the *Case Drop Stage*. Therefore, government departments should incorporate emotional valence into their operational policies, and pay greater attention to the role of negative emotions when public health emergencies occur, and make use of them when editing posts, so as to promote CEGSM and achieve efficient health communication.

Finally, our most outstanding contribution is exploration of influence factors of the CEGSM at the different stages of the COVID-19 crisis, and the role they play. The development of the crisis would lead to continuous changes in public psychology across stages. Governments must understand the differences of influencing factors on CEGSM at different stages, and timely adjust their response policies to achieve efficient health communication. This study provided a new perspective for CEGSM, that is, when public health emergencies occur, different crisis development stages also have an impact on CEGSM. Government agencies must divide crises into stages and analyze its development and public psychological needs at each stage (64, 65). Further, they must make rational and effective use of GSM to eliminate public anxiety, smash rumors and spread information.

### Limitations and Future Directions

This study has several limitations. First, although we adopt a commonly used approach to measure CEGSM (15, 66), several scholars have proposed other alternatives, for example some have explored the influencing factors of number of likes, reposts, and comments, respectively (15, 49). Future research can use alternative measures of CEGSM and compare them against this study. In addition, despite the popularity of government Sina Weibo accounts and the high citizen engagement, future citizen engagement and their influencing factors are likely to demonstrate more complexity, such as the possible novel user pattern of cross-platform and multi-stage crises. Future research can collect data from individuals and conduct typological research on their behaviors and distinguish the influencing factors of different types of citizen engagement. In addition, some variables may have better measurement, for example, word count may not be sufficient to reflect the completeness of the information. Sometimes a well-crafted concise message may contain more useful information than a redundant long message. Second, this study features the contributions of 22 local government official Sina Weibo accounts in high-risk areas of the Hubei province during the COVID-19 epidemic, which may overlook the interplay from social media accounts at the central government level. This study also did not analyze the differences between local governments and central government social media accounts in promoting CEGSM. Future research can further explore the similarities and differences between the central and local government social media practices in crisis communication. Third, although this study compared the differences of CEGSM at the different stages of the epidemic, the classification for the different stages of the epidemic came from the official declaration. For specific individuals, perceptions of the epidemic risks did not depend entirely on the epidemic stage defined by the officials. Future research can employ a tracing approach to observe variations in public perceptions and analyze the behaviors and differences of CEGSM against different backgrounds of the epidemic risk perception.

## Data Availability Statement

The raw data supporting the conclusions of this article will be made available by the authors, without undue reservation.

## Author Contributions

WZ, HY, CZ, QC, and RE conceived the study and developed the design. WZ, HY, and QC analyzed the result and supervised the study. WZ and HY wrote the first draft. CZ, RE, WZ, and QC revised the paper. All authors contributed to the article and approved the submitted version.

## Funding

This work was supported by National Natural Science Foundation of China (7210041729), the Fundamental Research Funds for the Central Universities (2021WKYXQN018), National Social Science Foundation of China (17CXW026), Natural Science Foundation of Shaanxi Province (Project No. 2022JM-418), and Social Science Foundation of Shaanxi Province (Project No. 2021ZXWT03).

## Conflict of Interest

The authors declare that the research was conducted in the absence of any commercial or financial relationships that could be construed as a potential conflict of interest.

## Publisher's Note

All claims expressed in this article are solely those of the authors and do not necessarily represent those of their affiliated organizations, or those of the publisher, the editors and the reviewers. Any product that may be evaluated in this article, or claim that may be made by its manufacturer, is not guaranteed or endorsed by the publisher.
